# The Role of 3D Printing in Endodontic Treatment Planning: A Comprehensive Review

**DOI:** 10.1055/s-0044-1791242

**Published:** 2024-11-07

**Authors:** Mustafa Hussein Alattas

**Affiliations:** 1 Department of Conservative Dental Sciences and Endodontics, College of Dentistry, Qassim University, Mulaidaa, Buraydah, Saudia Arabia

**Keywords:** 3D printing, endodontics, applications, treatments

## Abstract

This review aims to provide an overall picture of the three-dimensional (3D) printing contributions to endodontic practice in treatment planning and execution. The methodology entails a comprehensive literature review of the technological processes and 3D printing applications in the field. Some key findings show that 3D printing is highly effective in producing the right dental models for training, helps in complex surgeries, and supports the transition toward personalized therapies. The review reveals that 3D printing has many benefits but that the broader adoption of this technology faces issues, including high technical requirements, high costs, and the need for safety standards. The study concludes that although in the future some challenges need to be addressed, the potential of 3D printing in endodontics is enormous and this means that the treatment methods of dentistry could be more efficient and innovative.

## Introduction

Three-dimensional (3D) printing is a process of building up a 3D model created by using computer-aided design (CAD) software from an arrangement of successive two-dimensional (2D) layers. In terms of endodontic treatment planning, this is a paradigm shift in dental medicine to tailor treatment to each patient in a way that has not been possible before. This technology is important because it does not restrict imaging to only two dimensions which is a necessity in managing complex dental structures. 1 2 This review reflects the multidimensional nature of 3D printing in the practice of endodontics by illustrating how 3D printing is used in treatment planning, treatment delivery, and education. 3D printing has also been applied for the production of replicas of the root canal system that are high resolution for easier treatment planning in endodontics. 3 The unique features of this technology are especially useful in the creation of patient-specific models and guides for navigation through the root canal anatomy especially where the canals are blocked or have unusual anatomy. Its capability to promote clear imaging and planning not only helps in improving the final results of the treatment but also in reducing the risks and complications that is related to carrying out endodontic procedures. 4 5

Besides, 3D printing has transformed the training of endodontic practitioners. The shift toward the haptic simulators and printed models has been a great advancement for learning as it offers practical platforms on which students and practitioners can learn and improve their skills. 6 7 It is important in this respect to make the clinician prepared for the variety of complex situations that may be encountered during practice. Shaikh et al described the future of 3D printing in precise and individualized endodontic procedures. 8 It can be employed in the planning and execution of challenging medical procedures such as root canal treatments by facilitating the creation of individualized models. CAD/computer-aided manufacturing software with cone beam computed tomography (CBCT) and 3D printing also allows oral surgeons and endodontists to perform complex surgical procedures better and faster. But its use in education especially in the education of models used in the operative endodontic practice changed the nature of endodontic practice and the nature of endodontic education. 9

The reason for the need for 3D printing in endodontics is that 2D imaging is unable to provide an accurate representation of root canal morphology. 3D printing is therefore a better way of treating dental anatomy to understand the dental anatomy in detail to maximize the effectiveness of endodontic procedures, reducing chances of error, and increasing the chances of treatment success. 10 It also has the advantage of being an effective educational aid for the patients to understand their treatment and also improving the knowledge of dental professionals. The integration of 3D printing in endodontic treatment planning is thus a new and significant breakthrough as it helps deliver more precise, efficient, and personalized dental care.

## Methods

**Fig. 1:**
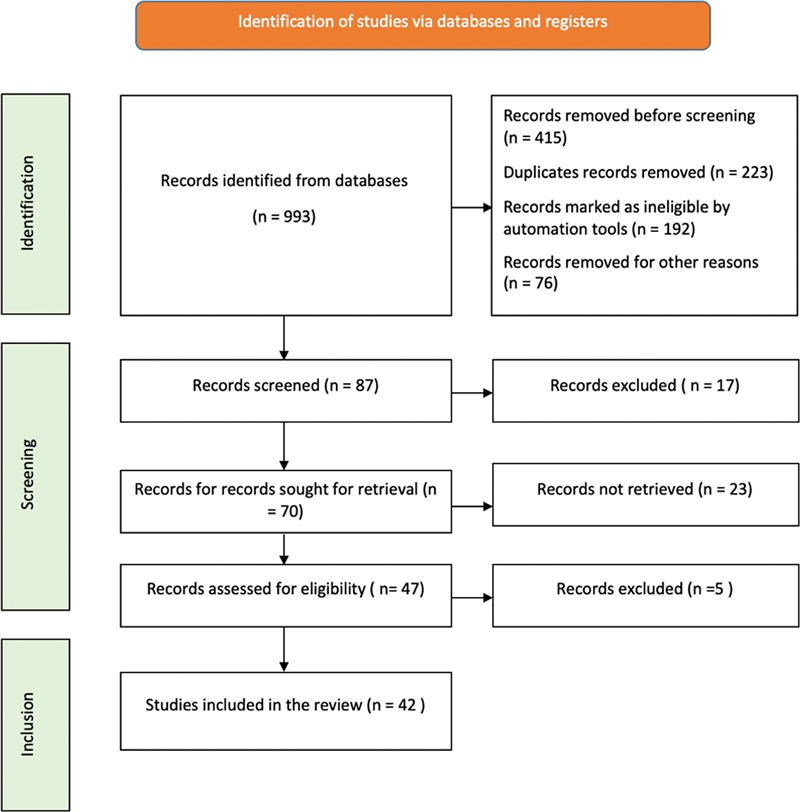
Flowchart showing the step-by-step identification of the studies via databases.

To conduct a literature survey on “The Role of 3D Printing in Endodontic Treatment Planning: Comprehensive Review,” a literature review was performed in March 2024 in electronic databases such as PubMed, Scopus, Embase, Cochrane Library, and ScienceDirect. The search was performed using MeSH terms/keywords: “3D printing,” “endodontic,” “treatment,” and “planning.” The electronic search was also complimented by cross-references and textbooks in a manual search for articles relevant to the subject. Exclusion criteria were articles that were not in the English language and were not published between March 2010 and March 2024. The process of article selection included evaluating inclusion and exclusion criteria and quality assessment. The initial sample included 993 articles but based on the titles and abstracts of the articles, 87 articles were selected for the review. After evaluating the full texts and applying the inclusion and exclusion criteria, 42 articles were chosen for the review, meeting the study's criteria ( Fig. 1 ).

## Historical Perspective

The paradigm shift in the endodontic treatment planning displays the shift from the traditional to the new and modern technologically savvy techniques. Bakland described these events by detailing how we evolved from employing methods such as the use of arsenicals to eliminate the cavity-causing “tooth worms” to the advanced techniques employed in cleaning, shaping, and filling the root canal system. 11 This evolution is characterized by major progress in the knowledge of etiology and diagnosis of pulpal and periapical pathology. 12 Patel added further explanation on these advancements and described the 3D printing and the CBCT for improved diagnosis and treatment planning. 13 The 3D root canal imaging was crucial to make sure that the information gathered reflected the actual anatomy in the treatment process to enhance the success rate. This evolution is a progressive step for endodontic treatments as it aims at preserving the natural dentition and improving the confidence and performance of patient-centered care. 14

It is also interesting to note that this historical retrospective of endodontics is relevant to such historical events in dentistry. This is as stated by Shah et al and Pauwels that the use of this 3D imaging machine such as CBCT helps in better diagnosis and treatment planning of the teeth and jaws. 15 16 They have facilitated the dental practitioners in understanding the intricate anatomy of the craniofacial region in a better way and do away with 2D radiography and adopt the more complicated form of imaging such as 3D imaging. They have not only led to increased diagnostic precision but have also significantly boosted the quality of care to patients. 17 18 Bozkurt and Karayel also explain how 3D printing developed and became more relevant in the field of medicine. 19 After the initial conceptualizations, 3D printing has been employed in different branches of medicine, including traditional applications and novel biomaterials, up to the emerging field of bioprinting. This evolution of technology with the ability to regenerate tissue and print live cells can be considered a promise of the future for the medical and dental fields, especially in the field of endodontics. 20

## 3D Printing Technologies

3D printing technologies have revolutionized several fields ranging from health care to aerospace sectors in the production of complex 3D objects from digital models. These techniques include stereolithography (SLA), fused deposition modeling (FDM), and selective laser sintering (SLS) which are suitable for various materials. 3D printing is a groundbreaking technology that has been used quite successfully for rapid prototyping (RP), producing customized products and complex geometries that would have been impossible to manufacture otherwise and it has been shaping the future of manufacturing, design, and material science.

Kafle et al published an article in 2021 which has given a complete review of 3D printing technologies which are being used in the field of dentistry specifically concentrating on FDM, SLS, and SLA. 21 FDM is the most popular method to fabricate polymer parts by which a thermoplastic material is melted and extruded as a part of the additive manufacturing. This method is one of the most widely used and relatively cost-effective techniques in the market today but has some drawbacks regarding high degree of resolution with possibility to see the layer effect. 22 SLS is another useful 3D printing technique where the layers of polymeric powder are painted and fused using a laser to form highly detailed and high-resolution parts for dentistry. However, it is more technically demanding and more expensive. SLA is the first 3D printing technology for dentistry, and it uses a photopolymer vat to cure objects using a light source thus forming intricate parts which are highly accurate but produces parts at a slower rate and requires further postprocessing. Every one of these technologies serves a critical function in dentistry from the production of dental models to the use of surgical guides and custom-made implants. 23 In studies by Pouhaër et al (2022) and Reymus et al (2019), the authors identified several materials that are used to 3D-printed endodontic models as well as their educational value. 24 25 Pouhaër et al studied the use of transparent liquid resin in the training of undergraduate teaching macromodels for large-scale models that contribute to the success of visualizing root canal anatomy and access cavity preparation. 26

On the other side, Reymus et al were interested in the possibility of using the SLA technology to manufacture artificial teeth for endodontic training. 27 Their study reveals that 3D photopolymers of photosensitive resin accurately replicate teeth and may be useful in preclinical education. 28 Behm et al outlined the benefits and the challenges associated with these 3D printing technologies. FDM is also one of the most studied and affordable for this reason. 12 SLS is more expensive, and hence, the 3D printing is of superior quality and needs expensive machinery. SLA has high accuracy and good surface finish, but it is relatively slower and requires postprocessing—it shows the comparative advantages and disadvantages of each technology. 29

## 3D Printing in Preclinical Endodontic Education

The use of 3D printing technology in preclinical endodontic education has greatly varied from the traditional model of teaching and learning. It gives the students a 3D view and visualization of the anatomies and also provides the students with 3D models of root canal configuration and practice and perfect their endodontic skills accurately and efficiently.

Studies about the effects of the use of 3D models in teaching and learning have been documented in the works of Smith et al, Ye et al, and Lim et al. 3 17 30 Smith et al underscored the fact that 3D printing dental models are useful in the development of students' understanding of the difficult aspects of the endodontics component, which is particularly important for the development of accurate clinical skills. 31 Ye et al conducted a systematic review and reported from meta-analyses of randomized controlled trials (RCTs) that 3D-printed models significantly enhance the accuracy and the speed of the students' answers which imply a better understanding of the subject. This was especially with regard to the study of neuroanatomy, cardiac anatomy, and abdominal anatomy as the use of 3D models when learning was far more effective when compared with other forms of learning. 32 Similarly, a RCT demonstrated that 3D-printed models could substitute cadaveric models for teaching the external cardiac anatomy and that the students' test results significantly improved after the substitution of 3D-printed models for cadaveric models. This is an implication that 3D models have superiority in some aspects of the learning of anatomy. 33

In addition, other research by Peters et al and Hanafi et al offered information on the relative benefits of 3D printing as a teaching method compared with conventional methods. 14 34 The studies found no significant improvement in the performance of root canal filling on 3D-printed models and identified that the use of 3D-printed models may not always benefit specific endodontic skills. 34 35 On the contrary, the study asserted that students viewed the modular 3D printing training models as more challenging yet perceived better prepared for the clinical setting and exhibited lower stress levels during subsequent clinical courses. 36 These conflicting results highlight the significance of 3D-printed models in enhancing the realism and efficacy of endodontic training in addition to indicating some of its disadvantages in contrast to conventional methods.

## Clinical Applications of 3D Printing in Endodontics

The innovation of 3D printing in endodontics has been a major breakthrough in the field of dental medicine following its increased precision and effectiveness in treatment. Its help in overcoming challenges such as negotiating calcified canals and periapical surgeries provides greater clinical efficacy while reducing endodontic procedures. Several research works have acknowledged 3D printing's transformative function in the clinical application of endodontics. 33 35 Some endodontic cases by Kamburoğlu et al that focused on 3D printing were calcified canals, periapical surgery, and autotransplantation. 35 From these three case studies, 3D printing's ability to acquire, design, and manufacture digital data was instrumental in reducing surgical time, clinical bias, and procedural failures. The case studies are a representation that increased 3D printing elevates the effectiveness and precision of handling endodontic cases. Balhaddad et al and Oberoi et al discussed the increased use of 3D printing across several dental disciplines and recommended the need for long-term study to understand the efficiency of these methods and setting safety thresholds in dental practice. They allude that more research is required to incorporate 3D printing in the endodontic practice fully. 33 37

Today, advanced case planning and simulation using 3D printing have changed the way endodontics are practiced by bringing accuracy and optimal individualized treatment. One of the essential applications of 3D printing in endodontics is the ability to create precise 3D models of patients' dental anatomy to plan and simulate endodontic treatment, such as root canal treatment or surgery. 5 Simulation in a 3D environment enables the comprehension of difficult cases and the production of a patient-customized surgical guide for optimal predictability and safety of endodontic therapies and the success of the treatment outcome. Another significant role of 3D printing in endodontics is the delivery of patient-specific endodontic appliances and guides, broadening the horizon of personalized medicine. 38 By using 3D-printed instruments, endodontic surgeries can be more precise and successful, reducing the likelihood of failure. Also, 3D has helped in enhancing endodontic surgery training by printing complex root canal anatomy, making it easier for the trainees to understand and practice the procedure hence better preparation for the clinical setting. 39

## Accuracy and Precision in 3D Printing for Endodontics

Now that the potential of 3D endodontics printing has been identified, it is worth noting that this field has changed considerably over the relatively short period. Accuracy and precision in 3D printing in endodontics are the key, as they make work on complex cases more efficient and successful. Specifically, the technology enables the creation of highly detailed and accurate replicas of dental structures. Corresponding anatomical forms help plan the necessary endodontic procedures more efficiently and, in the process, ensure they are performed with greater fidelity and exactness. The studies by Reymus et al and Pouhaër et al emphasized the dimensional accuracy of 3D-printed endodontic models and their dimensionality for dental education. 24 25 Reymus et al proved the feasibility of 3D printing of overall vigilant tooth replicas for endodontic skill development in dental students. 25 The trueness of the model was within 50.9 to 104.3 μm; the precision was even more precise and varied from 43.5 to 68.2 μm. Students acknowledged replicas profiting from their standardization and true-to-life appearance. 40 Pouhaër et al went further into 3D printing in dental education and demonstrated that the technology is formidable for endodontic procedures due to its dimensionality. The authors developed 3D macromodels of a lower first molar, which illustrated the root canal anatomy, and ideal access cavities. Due to their great size and transparency, student respondents noted that presented models were especially fruitful for understanding complex endodontic procedures. 41 Hence, 3D-printed models with accurate dimensional results contribute to endodontic education by promoting skill development and minimizing variations in preparation experiences.

Achieving high precision in endodontic treatment is critical, as it is closely connected with the results of the procedure and the ability to navigate and treat complex root canal systems in a highly accurate manner. 42 High-precision procedures not only help minimize procedural errors but also increase the treatment success rate at large, and enhance long-term patient comfort and overall dental health. Comparative analysis between traditional imaging and modeling techniques and more modern 3D printing methods shows a significant difference in terms of precision and detail exposure possible in endodontic treatment. 15 Traditional methods are still considered the most important ones, but these have been shown to only provide limited and 2D views that are inadequate to represent the 3D structure of a root canal system. 25 On the other hand, 3D printing, which is often based on advanced imaging such as CBCT, provides a better presentation of anatomy and thus better confidence in deciding upon the treatment approach or defining the precision of surgery. 34 Therefore, the increased use of 3D technology in endodontics has to be considered as the shift from approximate to precise and accurate treatments in cases of dental complexity.

## 3D Printing for Endodontic Research and Innovation

The technology of 3D printing has helped increase further development of endodontic research and innovations as it offers more precise tools to study intricate structures of the root canal system and create new opportunities for treatment. 43 Its use is vital for enhancing the current knowledge about the diseases linked to endodontic intervention and broadening the areas of research in an attempt to achieve new and more effective approaches in the field of dental hygiene. Studies on the topic conducted by Anderson et al and Pillai et al demonstrated that 3D printing is driving innovation and endodontic research. 4 29 Anderson et al demonstrated that 3D printing is being used for various endodontic applications, such as addressing access difficulties in root canals in pulp canal obliteration, autotransplant, presurgical planning, and educational modeling. 29 While most of these applications are only reported in a few case studies and preclinical studies, it is clear that they have the potential for the improve of the learning experience for students involved during practical instruction lectures. 19 Pillai et al and Connert et al highlighted that 3D printing is vital in making dental clinics transform from traditional to digital and that the technology is proven to be efficient, repeatable, and affordable due to the ability to produce treatment plans and models for educational purposes. 4 9 Both studies aimed at highlighting the impact that 3D printing technologies will have on the future of endodontic research, highlighting 3D printing to be the key area of development since it will enhance the overall experience both for students and patients by allowing a personalized approach to be used during those procedures.

The promising future of 3D printing in endodontics lies in the development of novel treatment approaches and materials. 31 3D printing techniques are likely to improve with the development of more advanced and biocompatible printing materials. This will allow for the production of customized endodontic devices and prosthetics which can be fitted inside a patient. 44 Moreover, the development and improvement of 3D printing techniques will improve treatment procedures, from complex endodontic surgery to regeneration therapies. This will result in more accurate, efficient, and minimally invasive treatment options. The emerging 3D bioprinting field in regenerative endodontics is a new development for restoring and healing dental tissue. 45 This state-of-the-art technique, which uses living cells and biomaterials to produce tissue-like structures, has the potential to radically improve dental pulp and periapical tissue regeneration. Bioprinting could have breakthroughs in the treatment of endodontic pathologies, providing a more natural solution to prevalent and ineffective traditional treatment options. 46

## Challenges and Limitations

While 3D printing has many advantages and potential in endodontics, it does come with various limitations and constraints. One of the most pressing technical obstacles to overcome is accurately producing 3D-printed models with precise details that replicate actual dental structures. Additionally, the challenges of endodontic practice in making knowledgeable decisions about acquiring skills necessary to implement advanced 3D printing technology are obtained from a practical standpoint. Finally, 3D printers will strive to make treatment production an easy option, in terms of cost and training, an easily doable solution to its limitations. 47

Another one of the factors that one can mention is the legal and regulatory factors. With 3D printing technology advancing at such an alarming rate, it is imperative that there be brought in and frequently updated a specific code of established standards and policies regarding the use of the technology in health care including endodontics. The usage of advancing technology must be highly safe, dentistry services continue to be at their best quality, and there is a sense of responsibility in the application of the technology in the clinical area. Meanwhile, the high cost, complexity, and the scale of regulation associated with 3D printing imply that it is a highly promising trend in terms of endodontic treatment and research that should be introduced to dental practice with great responsibility and regard for all aspects. 48

## Case Studies

The articles by Reis et al, Byun et al, Connert et al, Nosrat et al, and Gambarini et al offered useful information on the efficacy, advantages, and drawbacks of the use of 3D printing techniques in the endodontics. 9 18 28 38 49

Both Reis et al and Connert et al were concerned with the relevance and accuracy of 3D-printed models and guides for endodontic surgery. 9 38 Connert et al used a split-mouth design to compare traditional and guided endodontic American Dental Association–recommended techniques for access cavity preparation in teeth with calcified canals. The results achieved by the researchers showed that the 3D-printed-guided endodontics approach was superior in terms of successfully locating and navigating calcified root canals with significantly less dissection, less material removal, and faster treatment time. 9 Byun et al reported the efficacy of a 3D physical tooth model to treat a tooth anomaly. 28 This approach served to facilitate the diagnosis and management of the uncommon root canal anatomy, which highlights the strength of 3D printing in complex cases.

Nosrat et al and Gambarini et al focused on the more applied aspects of 3D printing in endodontics. 18 49 In terms of the creation of customized and biocompatible scaffolds for regenerative endodontics based on the patient-specific 3D printing, Gambarini et al explained the use of a dynamic navigation system in endodontic microsurgery—a new technique that was superior in its accuracy and prevented iatrogenic injuries from resulting from the procedure. 18 Habib and Sheikh evaluated the advantages of RP techniques in creating 3D-printed dental and biomedical implants, highlighting their benefits for patients previously untreatable with conventional methods. 41 Einbinder and Tobin (2022) assessed the current applications and effectiveness of 3D-printed guides in endodontics, finding that these technologies offer promising techniques with highly predictable outcomes and a low risk of iatrogenic damage, especially in complex cases. 42

Taken together, the above-mentioned studies demonstrate that 3D printing can generate accurate and efficient and potentially innovative endodontic treatments but also that expertise, costs, and continuing clinical trials are required for these new approaches.

## Conclusion

This extensive review showed how 3D printing is a powerful tool that can revolutionize endodontics by improving treatment planning, efficiency, and education. The benefits of 3D printing in endodontics are undeniable due to its increased precision, personalization, and practice-related simulations; however, there are challenges for its wider adoption. These include specific equipment and skills, expenses, and the changing legal and regulatory environments. These challenges can be addressed through higher education, technology innovations, and regulatory cooperation. There is no doubt that the technology has a bright future in endodontic practice and education as the technology advances and can potentially stay integrated into the field to provide innovative solutions to complex dental issues and completely change the face of endodontic treatment.
